# Association of serum potassium levels with mortality in critically ill patients with sepsis during hospitalization

**DOI:** 10.1371/journal.pone.0314872

**Published:** 2024-12-09

**Authors:** Guang Zhao, Yuting Gu, Yuyang Chen, Xiaohua Xia

**Affiliations:** Department of Emergency Medicine, Affiliated Kunshan Hospital of Jiangsu University, Kunshan, Jiangsu, China; Federal Medical Centre Umuahia, NIGERIA

## Abstract

**Background:**

Electrolyte disturbances are prevalent complications in critically ill patients with sepsis, significantly impacting patient prognosis. However, the specific association between serum potassium levels and mortality risk in this population remains poorly understood. This study aimed to investigate the association between serum potassium levels during hospitalization and the risk of 28-day and 90-day mortality in critically ill patients with sepsis.

**Methods:**

Data were obtained from the Medical Information Mart for Intensive Care (MIMIC-IV) database, and patients with severe sepsis requiring ICU admission were stratified into quartiles based on their mean serum potassium levels. Outcomes assessed included 28-day and 90-day mortality. A multivariate Cox proportional hazards model was used to investigate the association between serum potassium levels and mortality, with restricted cubic splines to identify potential nonlinear correlations. A dichotomous Cox proportional hazards model was applied to analyze the association further, and Kaplan-Meier analysis assessed the mortality risk across different potassium ranges.

**Results:**

A total of 25,203 patients were included, with 28-day and 90-day mortality rates of 27.84% and 40.48%, respectively. Multivariate analysis showed a significant association between serum potassium levels and mortality. Restricted cubic splines identified an inflection point at 4.4 mmol/L, with potassium levels above this threshold associated with higher mortality (28-day mortality: HR 2.96, 95% CI = 2.43–3.60; 90-day mortality: HR 2.19, 95% CI = 1.81–2.64). Kaplan-Meier analysis confirmed a significantly higher risk of death for patients with serum potassium levels above 4.4 mmol/L compared to those within the 3.5–4.4 mmol/L range (P<0.001).

**Conclusion:**

In critically ill patients with sepsis, serum potassium levels exceeding 4.4 mmol/L are associated with an increased risk of death. Maintaining the average serum potassium level within the range of 3.5–4.4 mmol/L appears to be safe and may contribute to better outcomes in this patient population.

## Introduction

Sepsis, characterized by life-threatening organ dysfunction resulting from a dysregulated host response to infection, stands as a primary cause of mortality among intensive care unit (ICU) patients, with approximately 20–30% succumbing to the condition within hospital walls [1, 2]. Despite extensive research efforts, including initiatives like the Sepsis Survival Campaign and the implementation of national core measures, sepsis mortality rates persist stubbornly high [3, 4]. Recognized as a syndrome rather than a distinct disease entity, sepsis involves complex abnormalities across multiple body systems and functions, encompassing cardiovascular, neuronal, metabolic, coagulation, and immune responses [5]. Consequently, predicting the clinical outcomes of ICU patients grappling with sepsis has emerged as a crucial research endeavor.

Electrolyte disturbances are prevalent among critically ill ICU patients and not only influence treatment strategies and ICU length of stay but also significantly impact patient prognosis. While researchers have extensively studied the effects of elevated sodium, increased phosphorus, and decreased calcium on clinical outcomes in patients with sepsis, serum potassium has received comparatively less attention [6–8]. Potassium, a vital electrolyte in the human body, plays a crucial role in maintaining the normal function of the cardiovascular system, skeletal muscle, visceral organs, and nervous system [9, 10]. Imbalances in serum potassium can lead to severe complications in critically ill patients, including arrhythmias and muscle weakness, with potential consequences such as cardiac arrest and respiratory failure [11]. In this study, we aimed to assess the predictive capacity of mean serum potassium levels for mortality in critically ill patients with sepsis and explore its association with sepsis-related mortality.

## Methods

### Study population

This study utilized health-related data obtained from the MIMIC-IV (version 2.2) repository, a comprehensive database managed by the MIT Computational Physiology Laboratory [12]. Researcher Guang Zhao adhered to all access protocols and conducted the data extraction. Patients diagnosed with sepsis according to the sepsis 3.0 diagnostic criteria were included [13]. Specific exclusion criteria were applied, including: (1) individuals below 18 years old at their initial admission; (2) patients with multiple sepsis admissions, with data extracted solely from the first admission; and (3) patients lacking adequate data, particularly serum potassium levels. A total of 25,203 patients were included in the analysis and stratified into four groups based on quartiles of serum potassium levels.

### Ethics statement

This study adhered to the regulations outlined in the Helsinki Declaration. Approval for accessing the MIMIC-IV database was granted by the review committee of Beth Israel Deaconess Medical Center and Massachusetts Institute of Technology. As the data is openly accessible within the MIMIC-IV database, the need for ethical approval and informed consent was waived in this study.

### Data collection

Various demographic and health-related factors were collected from the MIMIC-IV database and categorized into two primary domains: (1) Baseline characteristic variables, including age, race, sex, BMI, marital status, and comorbidities such as hypertension, diabetes, heart failure, renal failure, respiratory failure, and malignant tumor. (2) Clinical parameters, encompassing hemoglobin (Hb), platelet count (PLT), red blood cell count (RBC), hematocrit (HCT), white blood cell count (WBC), blood glucose, potassium (K^+^), sodium (Na^+^), serum calcium (Ca^2+^), albumin, lactate, and severity of illness scores such as Sequential Organ Failure Assessment (SOFA) score, Logistic Organ Dysfunction System (LODS) score, Simplified Acute Physiology Score II (SAPS-II), Oxford Acute Severity of Illness Score (OASIS), and Acute Physiology Score III (APSIII) [14–16]. Follow-up commenced on the day of admission and concluded on the day of death. With the exception of serum potassium, all laboratory variables and disease severity scores were extracted from data collected within 24 hours of patient admission.

### Outcomes

The primary endpoint of the study was 28-day mortality, with the secondary endpoint being 90-day mortality.

### Statistical analysis

Participants were stratified into four groups based on quartiles of mean potassium levels (Q1-Q4). Continuous variables were expressed as mean ± standard deviation (SD) or median (interquartile range, IQR) and compared using t-tests or Kruskal-Wallis rank sum tests, as appropriate. Categorical variables were presented as frequencies and percentages and compared using Fisher’s exact test when necessary. We assessed 28-day mortality and 90-day mortality for each potassium quartile during the follow-up period. A multivariate Cox proportional hazards regression model was employed to adjust for confounding factors and assess the independent predictive value of potassium. Model 1 remained unadjusted, while Model 2 included adjustments for age, sex, BMI, race, and marital status. Model 3 further incorporated adjustments for hypertension, diabetes, heart failure, renal failure, respiratory failure, and cancer based on Model 2. To explore the nonlinear relationship between potassium ion and mortality, we utilized restricted cubic spline Cox proportional hazards regression models and smooth curve fitting (penalized spline). The threshold was estimated by evaluating all possible values and selecting the most probable threshold point. We investigated the association between potassium and 28-day and 90-day mortality risk using two-piecewise Cox proportional hazards models on either side of the inflection point. Additionally, we conducted stratified analyses by sex, age (<70 years or ≥70 years), race, marital status, and coexisting conditions to assess the consistency of potassium’s prognostic value on outcomes. Furthermore, we employed Kaplan-Meier curves to analyze the risk of death in relation to serum potassium levels. Statistical significance was defined as two-sided p-values less than 0.05. All statistical analyses were performed using IBM SPSS version 24.0 and Empowerstats software version 6.0.

## Results

### Baseline characteristics

Table 1 displays the baseline characteristics of the cohort study participants (n = 25,203), categorized by serum potassium quartile. The mean age of participants was 67.3 years, with 57.79% being male. When compared to participants in the lowest quartile, those with higher potassium levels were more likely to be male, have diabetes, heart failure, and renal failure, and exhibited higher rates of in-hospital and 28-day mortality. Additionally, significant differences were observed in biochemical parameters between the groups. Participants in the highest quartile demonstrated significantly elevated platelet (PLT), blood glucose, and calcium (Ca^2+^) levels, as well as higher Sequential Organ Failure Assessment (SOFA) score, Simplified Acute Physiology Score II (SAPS II), and Acute Physiology Score III (APS III) scores compared to those in the first quartile. However, levels of albumin, lactate, and mean arterial pressure (MAP) were notably lower than those in the first quartile.

**Table 1:** Baseline characteristics according to the serum potassium quartiles.

	Quartiles of potassium					P value
	overall	Q1	Q2	Q3	Q4	
		(0.9–3.7)	(3.8–4)	(4.1–4.4)	(4.5–16.7)	
N	25203	6223	5014	7469	6497	
Age, years	67.30	65.30	66.70	68.10	68.70	<0.001
	(56.10–78.20)	(53.30–77.20)	(55.23–78.50)	(57.30–78.40)	(58.40–78.80)	
BMI, kg/m2	27.95	27.43	27.76	27.92	28.56	<0.001
	(24.26–32.62)	(23.51–32.50)	(24.19–32.26)	(24.43–32.28)	(24.85–33.21)	
Gender						<0.001
female	10639 (42.21%)	3187 (51.21%)	2270 (45.27%)	2887 (38.65%)	2295 (35.32%)	
male	14564 (57.79%)	3036 (48.79%)	2744 (54.73%)	4582 (61.35%)	4202 (64.68%)	
Race						<0.001
white	16832 (66.79%)	4059 (65.23%)	3333 (66.47%)	5087 (68.11%)	4353 (67.00%)	
black	2169	566	378	575	650	
	(8.61%)	(9.10%)	(7.54%)	(7.70%)	(10.00%)	
others	6202 (24.61%)	1598 (25.68%)	1303 (25.99%)	1807 (24.19%)	1494 (23.00%)	
Marital status						<0.001
single	6561 (28.74%)	1754 (31.35%)	1400 (31.08%)	1792 (26.32%)	1615 (27.26%)	
married	11422 (50.03%)	2604 (46.54%)	2146 (47.64%)	3598 (52.85%)	3074 (51.89%)	
divorced	1785	480	358	513	434	
	(7.82%)	(8.58%)	(7.95%)	(7.54%)	(7.33%)	
widowed	3064 (13.42%)	757	601	905	801	
		(13.53%)	(13.34%)	(13.29%)	(13.52%)	
Hb (g/dL)	11.7	11.7	11.7	11.8	11.6	<0.001
	(10.0–13.3)	(10.0–13.2)	(10.0–13.3)	(10.2–13.4)	(10.0–13.2)	
PLT (K/uL)	211 (153–280)	204 (145–274)	210 (152–276)	212 (156–277)	216 (156–290)	<0.001
RBC (m/uL)	3.84 (3.27–4.38)	3.83 (3.29–4.35)	3.86 (3.28–4.40)	3.87 (3.30–4.39)	3.79 (3.24–4.36)	<0.001
HCT (%)	35.4 (30.7–39.8)	34.9 (30.3–39.2)	35.2 (30.5–39.7)	35.9 (31.2–40.0)	35.4 (30.6–40.0)	<0.001
WBC (K/uL)	9.6 (6.9–13.6)	9.7 (6.8–13.8)	9.8 (6.9–13.8)	9.5 (7.0–13.3)	9.6 (7.0–13.5)	0.294
Glucose (mg/dL)	120 (100–154)	119 (100–150)	121 (101–155)	119 (100–152)	120 (99–159)	0.121
Na+ (mmol/L)	139 (136–141)	139 (136–141)	139 (136–141)	139 (136–141)	138 (135–140)	<0.001
Ca2^+^(mmol/L)	1.11 (1.05–1.16)	1.08 (1.02–1.14)	1.10 (1.05–1.16)	1.12 (1.07–1.17)	1.13 (1.07–1.18)	<0.001
Albumin (g/dL)	3.4 (2.8–3.9)	3.3 (2.8–3.8)	3.3 (2.8–3.8)	3.5 (2.9–3.9)	3.4 (2.9–3.9)	<0.001
Lactate (mmol/L)	1.7 (1.2–2.5)	1.8 (1.3–2.65)	1.7 (1.2–2.5)	1.6 (1.2–2.3)	1.6 (1.2–2.5)	<0.001
MAP (mmHg)	77 (67–90)	80 (69–92)	79 (69–92)	76 (67–87)	75 (65–88)	<0.001
SOFA	3 (2–4)	3 (2–4)	3 (2–4)	3 (2–4)	3 (2–5)	<0.001
LODS	5 (3–7)	5 (3–7)	5 (3–7)	5 (3–7)	5 (3–8)	<0.001
SAPSII	37 (29–46)	35 (27–44)	36 (28–44)	36 (29–46)	40 (32–51)	<0.001
OASIS	33 (27–39)	33 (27–39)	33 (27–39)	32 (27–38)	33 (28–39)	<0.001
APSIII	45 (33–60)	44 (33–59)	44 (32–59)	43 (32–58)	47 (35–64)	<0.001
**Commorbidities**						
Hypertension						<0.001
no	13881 (55.08%)	3324 (53.41%)	2745 (54.75%)	4028 (53.93%)	3784 (58.24%)	
yes	11322 (44.92%)	2899 (46.59%)	2269 (45.25%)	3441 (46.07%)	2713 (41.76%)	
Diabetes						<0.001
no	17609 (69.87%)	4777 (76.76%)	3636 (72.52%)	5175 (69.29%)	4021 (61.89%)	
yes	7594 (30.13%)	1446 (23.24%)	1378 (27.48%)	2294 (30.71%)	2476 (38.11%)	
Heart Failure						<0.001
no	19665 (78.03%)	5158 (82.89%)	3976 (79.30%)	5657 (75.74%)	4874 (75.02%)	
yes	5538 (21.97%)	1065 (17.11%)	1038 (20.70%)	1812 (24.26%)	1623 (24.98%)	
Kidney Failure						<0.001
no	12627 (50.10%)	3332 (53.54%)	2660 (53.05%)	3907 (52.31%)	2728 (41.99%)	
yes	12576 (49.90%)	2891 (46.46%)	2354 (46.95%)	3562 (47.69%)	3769 (58.01%)	
Respiratory Failure						<0.001
no	15533 (61.63%)	3791 (60.92%)	2995 (59.73%)	4727 (63.29%)	4020 (61.87%)	
yes	9670 (38.37%)	2432 (39.08%)	2019 (40.27%)	2742 (36.71%)	2477 (38.13%)	
Malignant tumor						0.089
no	17520 (69.52%)	4349 (69.89%)	3537 (70.54%)	5188 (69.46%)	4446 (68.43%)	
yes	7683 (30.48%)	1874 (30.11%)	1477 (29.46%)	2281 (30.54%)	2051 (31.57%)	
**Events**						
Death in hospital						<0.001
no	22717 (90.14%)	5678 (91.24%)	4547 (90.69%)	6845 (91.65%)	5647 (86.92%)	
yes	2486 (9.86%)	545 (8.76%)	467 (9.31%)	624 (8.35%)	850 (13.08%)	
28-day mortality						<0.001
no	7777 (72.16%)	1937 (72.68%)	1523 (73.72%)	2220 (75.38%)	2097 (67.62%)	
yes	3000 (27.84%)	728 (27.32%)	543 (26.28%)	725 (24.62%)	1004 (32.38%)	
90-day mortality						0.024
no	6414 (59.52%)	1598 (59.96%)	1229 (59.49%)	1805 (61.29%)	1782 (57.47%)	
yes	4363 (40.48%)	1067 (40.04%)	837 (40.51%)	1140 (38.71%)	1319 (42.53%)	

BMI, body mass index; Hb, hemoglobin; PLT, platelet; RBC, red blood cell; HCT, hematocrit; WBC, white blood cell; SOFA, sequential organ failure assessment; LODS, logistic organ dysfunction system score; OASIS, oxford acute severity of illness score; SAPSII, simplified acute physiology score ii

### Relationships of potassium with mortality

Multivariable Cox regression analysis was utilized to construct three models (Table 2). In Model 3, following adjustments for age, sex, BMI, race, marital status, hypertension, diabetes, heart failure, renal failure, respiratory failure, and malignancy, serum potassium levels were evaluated in quartiles relative to Q1 (reference). We observed hazard ratios (HRs) for potassium and 28-day mortality for Q2, Q3, and Q4 to be 0.73 (95% CI: 0.59, 0.91, P = 0.0044), 0.78 (95% CI: 0.64, 0.95, P = 0.0141) and 1.10 (95% CI:0.92,1.32, P = 0.3074), respectively. For 90-day mortality, the HRs were 0.70 (95% CI: 0.57, 0.85, P = 0.0003) and 0.72 (95% CI: 0.60, 0.86, P = 0.0003) for Q2 and Q3, respectively. Notably, there was a nonlinear relationship between serum potassium levels and both 28-day and 90-day mortality.

**Table 2:** Hazard ratios (95% CI) for mortality based on potassium quartiles.

Varible	Crude		Mode I		Model II	
	HR (95% CI)	P value	HR (95% CI)	P value	HR (95% CI)	P value
**28-day mortality**						
Q1	1 (ref)		1 (ref)		1 (ref)	
Q2	0.70 (0.62, 0.81)	<0.0001	0.72 (0.58, 0.88)	0.002	0.73 (0.59, 0.91)	0.0044
Q3	0.68 (0.60, 0.77)	<0.0001	0.75 (0.62, 0.91)	0.0035	0.78 (0.64, 0.95)	0.0141
Q4	1.06 (0.95, 1.19)	0.2919	1.03 (0.87, 1.23)	0.7264	1.10 (0.92, 1.32)	0.3074
**90-day mortality**						
Q1	1 (ref)		1 (ref)		1 (ref)	
Q2	0.70 (0.62, 0.79)	<0.0001	0.69 (0.57, 0.84)	0.0001	0.70 (0.57, 0.85)	0.0003
Q3	0.69 (0.61, 0.77)	<0.0001	0.70 (0.59, 0.84)	0.0001	0.72 (0.60, 0.86)	0.0003
Q4	1.05 (0.95, 1.17)	0.3511	1.04 (0.89, 1.23)	0.6003	1.08 (0.91, 1.27)	0.3908

Adjust model I adjust for: age, sex, BMI, race, and marital status

Adjust model II adjust for: age, sex, BMI, race, marital status, hypertension, diabetes, heart failure, renal failure, respiratory failure, and malignancy

### The detection of nonlinear relationships

**Fig 1:**
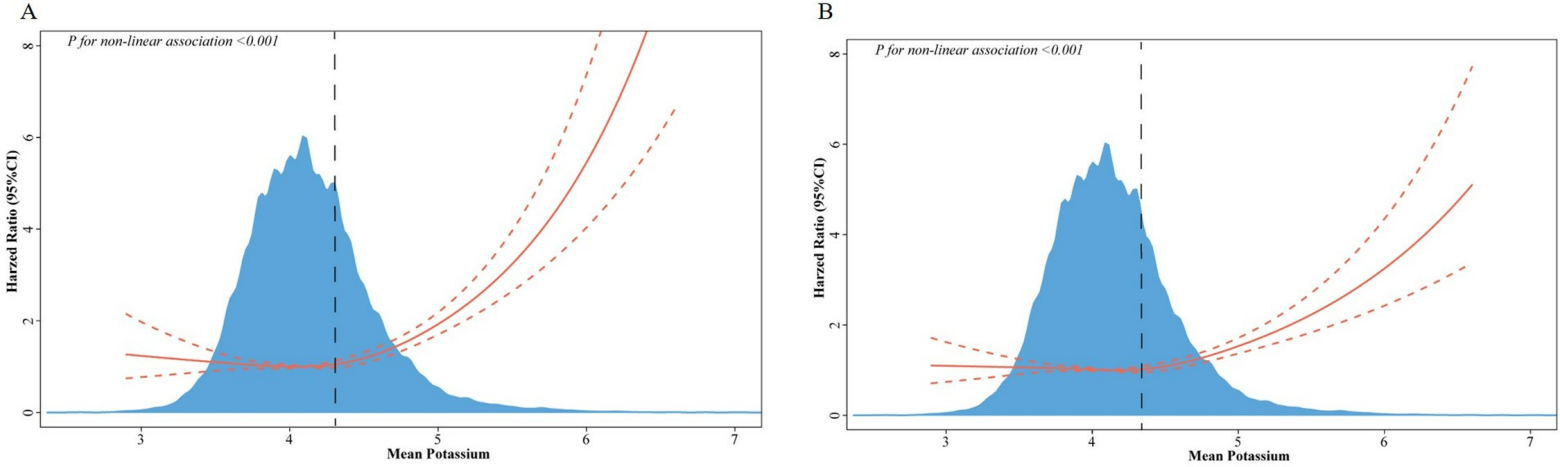
Association of serum potassium levels with mortality. Association of serum potassium levels with 28-day mortality (A) and 90-day mortality (B) in septic patients, with each hazard ratio calculated using a potassium level of 4.4 as a reference for both (A) and (B). The analysis is adjusted for age, sex, BMI, race, marital status, hypertension, diabetes, heart failure, renal failure, respiratory failure, and malignancy. Solid lines represent the hazard ratio estimates, while the red areas depict their corresponding 95% confidence intervals.

Cox proportional hazards regression models employing restricted cubic splines and smooth curve-fitting (penalized splines) were utilized to delve deeper into the association, building upon multivariate Cox regression analyses revealing a nonlinear relationship between serum potassium levels and both 28-day and 90-day mortality. Adjusted smoothed plots displayed a discernible upward trend, illustrating the association between serum potassium levels and both 28-day (Fig 1A) and 90-day mortality (Fig 1B). Furthermore, standard Cox proportional-hazards regression models and two-segment Cox proportional-hazards regression models were fitted to analyze the association between baseline potassium concentrations and mortality. Employing a two-segment Cox proportional hazards regression model, an inflection point of 4.4 was determined for both 28-day and in-hospital mortality (both P-values log-likelihood ratio <0.001) (Table 3). Upon adjustment for various covariates, the risk of both 28-day and 90-day mortality increased significantly by 196% per unit increase in potassium concentration (HR 2.96, 95% CI = 2.43–3.60, P < 0.01) and 119% (HR 2.19, 95% CI = 1.81–2.64, P < 0.0001), respectively.

**Table 3:** Threshold effect analysis of potassium on 28-day mortality and 90-day mortality in patients with sepsis.

	Adjusted
	HR (95%CI) / P value
**28-day mortality**	
Fitting by two-piecewise Cox proportional risk model	
inflection point	4.4
K < 4.4	0.95 (0.77, 1.17) 0.6138
K > = 4.4	2.96 (2.43, 3.60) <0.0001
P for Log-likelihood ratio	<0.001
**in-hospital mortality**	
Fitting by two-piecewise Cox proportional risk model	
inflection point	4.4
K < 4.4	0.91 (0.76, 1.08) 0.2796
K > = 4.4	2.19 (1.81, 2.64) <0.0001
P for Log-likelihood ratio	<0.001

Adjust model adjust for: age, sex, BMI, race, marital status, hypertension, diabetes, heart failure, renal failure, respiratory failure, and malignancy.

### Stratified analyses

**Fig 2:**
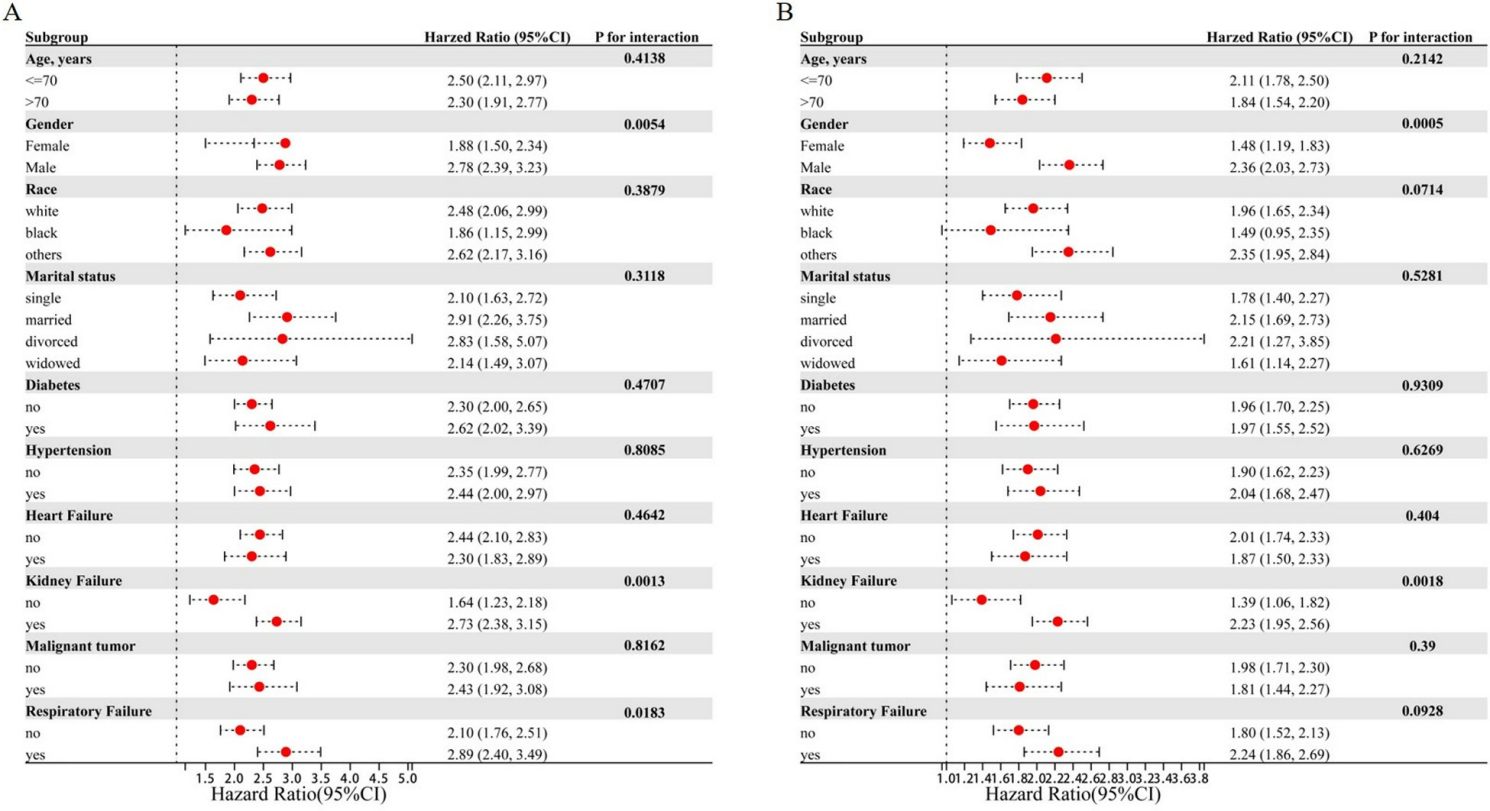
Stratified analyses of the associations between potassium and mortality. Stratified analysis of serum potassium levels and their association with 28-day mortality (A) and 90-day mortality (B). Serum potassium exhibited consistent associations across subgroups for both 28-day and 90-day mortality, except for the male and renal failure subgroups.

To further evaluate the relationship between serum potassium levels and the risk of 28-day mortality (Fig 2A) and 90-day mortality (Fig 2B) across various subgroups, we conducted stratified analyses based on age, sex, race, marital status, diabetes, hypertension, heart failure, renal failure, respiratory failure, and malignancy. Our findings revealed that both 28-day and 90-day mortality risks were notably higher in male patients compared to their female counterparts. Similarly, individuals with renal failure exhibited significantly elevated mortality risks compared to those without renal failure. Otherwise, serum potassium demonstrated consistent associations across most subpopulations.

### Impact of serum potassium within the physiological range on the mortality risk

**Fig 3:**
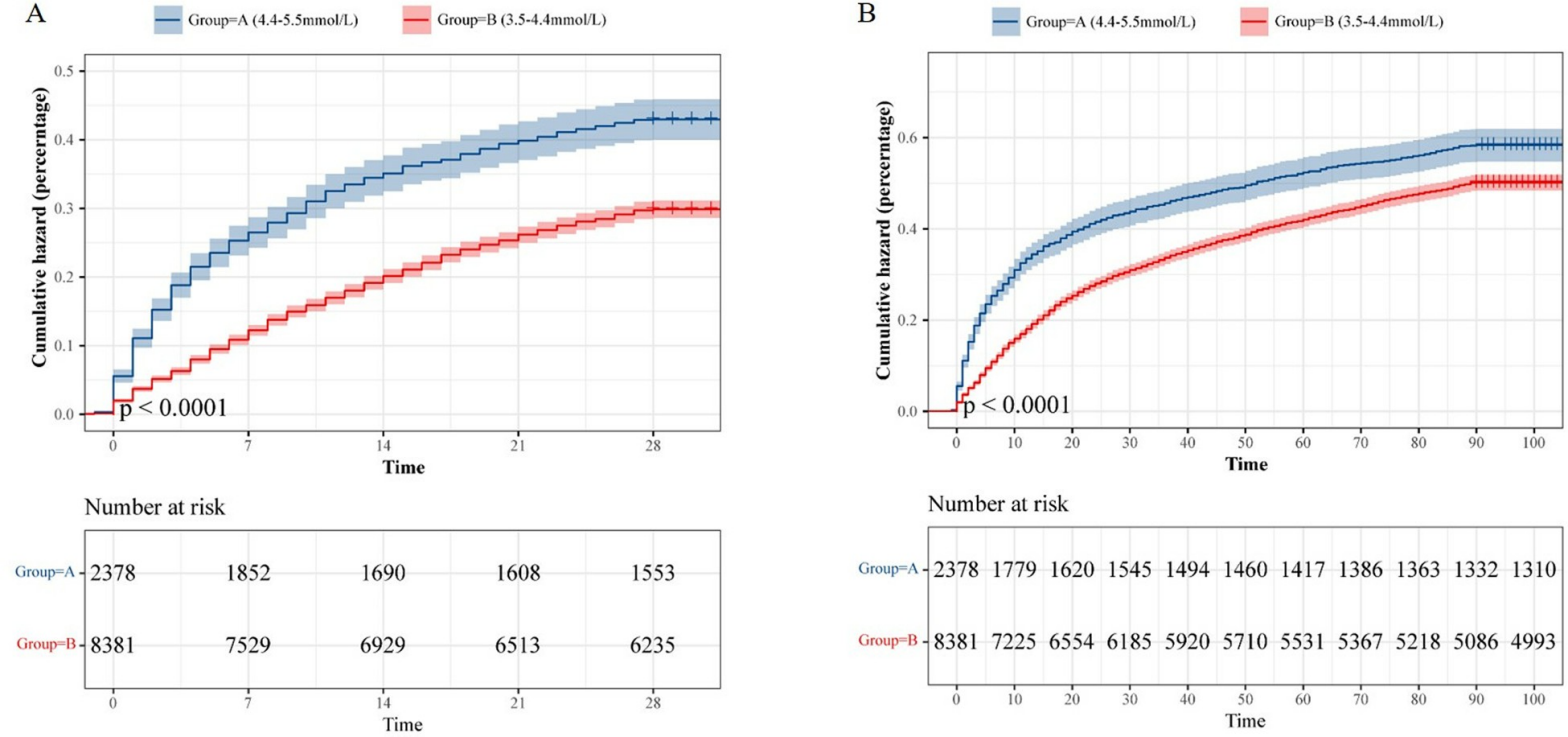
Kaplan-Meier curves for 28-day and 90-day cumulative mortality risk. The cumulative risk of 28-day (A) and 90-day mortality (B) in critically ill patients with sepsis stratified by serum potassium levels of 3.5–4.4mmol/L and 4.4–5.5mmol/L.

In this study, we observed a gradual increase in the risk of death associated with serum potassium levels exceeding 4.4mmol/L. To explore potential differences in mortality risk within the normal range of serum potassium, we categorized potassium levels into two groups: 3.5–4.4mmol/L and 4.4–5.5mmol/L. Kaplan-Meier analyses were conducted to compare the risks of death at 28 days (Fig 3A) and 90 days (Fig 3B) between these groups. Our findings revealed that patients with higher serum potassium levels had a significantly elevated risk of death compared to those with lower serum potassium levels (P<0.001).

## Discussion

This study marks the inaugural investigation into the association between mean serum potassium levels and mortality among patients with severe sepsis. Through threshold effect analysis, we identified that mortality risk in septic patients exhibited a curvilinear increase when mean serum potassium exceeded 4.4mmol/L. This underscores the robust predictive value of serum potassium for both 28-day and 90-day mortality outcomes. Additionally, maintaining serum potassium levels within the range of 3.5–4.4mmol/L appears to be a safe and prudent approach.

During ICU stays, critically ill patients frequently experience electrolyte disturbances, significantly correlating with elevated mortality rates [17, 18]. Electrolytes play pivotal roles in various physiological functions, with precise intracellular and extracellular concentrations crucial for normal metabolic processes and organ function [19, 20]. The electrolyte profiles of critically ill patients manifest considerable heterogeneity due to disease status and specific interventions like fluid resuscitation and drug therapy, complicating electrolyte disorder correction and treatment strategies [21–23]. Potassium ions notably play a critical role, with deviations in blood potassium levels disrupting normal physiological function, impacting patient prognosis, and potentially precipitating organ failure in severe cases. Hypokalemia can induce acidosis, renal dysfunction, muscle cramps, and arrhythmias, while hyperkalemia can precipitate muscle paralysis, cardiac arrest, or life-threatening arrhythmias [24, 25]. Studies consistently demonstrate that both hypokalemia and hyperkalemia heighten mortality risk, with these electrolyte abnormalities closely associated with elevated risks of fatal complications [26]. The observed association between serum potassium levels and mortality risk in critically ill patients with sepsis underscores the importance of monitoring and managing electrolyte imbalances in this population.

Sepsis, a prevalent critical condition in the ICU, requires close attention to its clinical manifestations, particularly hypotension and shock, which signify more severe stages of the illness. The elevation of blood potassium levels correlates with diminished blood pressure. Intracellular potassium ion efflux precipitates persistent vasodilation, further exacerbating hypotension [27]. Additionally, potassium efflux induces continuous hyperpolarization of cell membranes, resulting in impaired vascular reactivity in septic patients [28]. This elucidates why the administration of vasopressors does not significantly ameliorate microcirculatory disorders [29]. Potassium ion imbalance also escalates the risk of arrhythmias and potentially heart failure, thereby amplifying mortality risks in critically ill septic patients [30]. Research has substantiated the involvement of potassium ions in macrophage transmembrane signal transduction, eliciting cytokine production, and fostering inflammatory responses [31]. Consequently, vigilant monitoring of potassium level fluctuations not only mitigates complications in septic patients but also curtails inflammation progression, thereby improving patient prognoses.

In our investigation, we observed a considerable elevation in mortality risk associated with potassium levels exceeding 4.4mmol per liter. Consequently, we sought to explore whether this heightened mortality risk persisted among septic patients with serum potassium levels within the physiological normal range (3.5 to 5.5mmol/L). Utilizing Kaplan-Meier analysis, we discerned a noteworthy reduction in mortality risk when patients maintained lower normal potassium levels. This finding aligns with a substantial retrospective observational study, which identified potassium levels exceeding 4.5 mmol/L as a robust independent predictor of 30-day all-cause mortality, with mortality risk escalating alongside potassium levels [32, 33]. Moreover, Hessels et al. [26]suggested that maintaining serum potassium concentrations between 3.5 and 4.0 mmol/L was safe for critically ill patients. Building upon these insights, we advocate for maintaining potassium levels within the safe range of 3.5–4.4mmol/L in critically ill patients with sepsis.

This study possesses several limitations. Firstly, as an observational study conducted at a single center, it cannot establish causality. Despite employing multivariate adjustment to control for confounding variables, the potential for residual confounding impacting prognosis remains. Moreover, the absence of personal patient histories, such as smoking and alcohol consumption, represents another limitation.

## Conclusion

In conclusion, our findings suggest that mean serum potassium levels are a valuable predictor of 28-day and 90-day mortality risk in critically ill patients with sepsis, with a safe target range identified between 3.5 and 4.4 mmol/L. Therefore, assessing mean serum potassium levels during hospitalization may provide insight into risk assessment and prognosis within this patient population. Future research endeavors should aim to investigate the effectiveness of interventions aimed at modulating serum potassium levels to improve clinical outcomes in these individuals.
